# Artificial optical synaptic devices with ultra-low power consumption

**DOI:** 10.1038/s41377-022-01066-2

**Published:** 2023-01-16

**Authors:** Guoqiang Li

**Affiliations:** State Key Laboratory of Luminous Materials and Devices, South China University of Technology, Guangzhou, 510641 China

**Keywords:** Photonic devices, Bioinspired materials

## Abstract

A BP/CdS heterostructure-based artificial photonic synapse with an ultra-low power consumption is proposed, presenting great potential in high-performance neuromorphic vision systems.

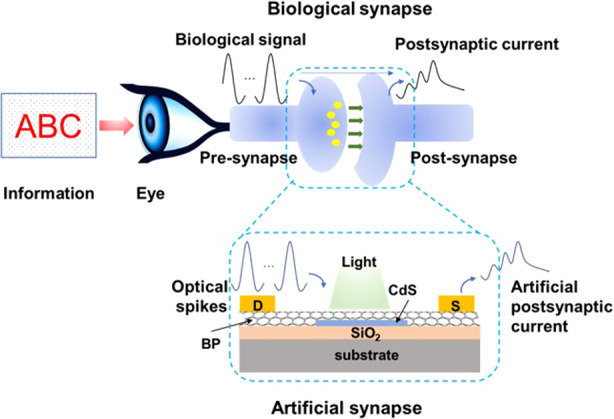

## Abstract (web summary)

A BP/CdS heterostructure-based artificial photonic synapse with an ultra-low power consumption is proposed, presenting great potential in high-performance neuromorphic vision systems.

With the rise of the Internet big data and the explosive growth of information, artificial intelligence (AI) system has entered a new period of rapid development and gets considerable attention^1^. Logical computing based on the von Neumann structure cannot meet the needs of the Internet of Things (IoT), edge computing, because the architecture of memory and processor separation not only increases the time to process information, but also causes huge energy consumption. Compared with traditional computer systems, artificial neural networks can mimic the basic functions of human brain neurons and perform efficient data processing in a distributed, parallel and event-driven manner, which is an important means to break the “von Neumann bottleneck” and realize a new generation of artificial intelligence computer^2^.

Artificial synaptic devices are one of the core hardware of neuromorphic computing systems, which have abilities to reveal massively parallel, efficient and high-speed, and handle non-structural problems^3^. Compared with electronic synaptic devices, photonic synaptic devices are driven by optical signals to realize synaptic functions, which have the advantages of fast, high bandwidth, low crosstalk and low power consumption^4,5^. More importantly, photonic synaptic devices respond to and process light signals directly and can process visual and image information directly, so they can be used to simulate the function of human retina. If combined with other neural components, they can form a visual neural network and realize an artificially simulated neuromorphic visual system. Therefore, developing a new type of high-performance photonic synaptic device with “storage and computation integration” is important to realize an artificial intelligence system based on a photonic neural network^6–8^.

Different artificial synapse prototypes have been successfully constructed in recent years based on low-dimensional materials^9^, organic materials^10,11^, and perovskites^12,13^. In 2015, Kang et al.^9^ investigated the effect of self-assembled monolayer (SAM)-based doping on the performance of WSe_2_- and MoS_2_- based transistors and photodetectors, the responsibility reached 1.45 × 10^4 ^A/W. Deng et al.^10^ demonstrated a new organic photosynaptic device in 2019, which featured synaptic and optical-sensing functions in a single device. This device successfully avoided the use of sophisticated device architectures and had a power consumption of 150 μW/cm^2^. In 2021, Zhu et al.^13^ presented a flexible optoelectronic sensor array of 1024 pixels using a combination of carbon nanotubes and perovskite quantum dots as active materials for an efficient neuromorphic vision system. The device exhibited a high sensitivity to light of 5.1 × 10^7 ^A/W. However, photonic synapse, one of the key components, is still suffering high power consumption, potentially limiting its applications in an artificial neural system.

Now, writing in this issue of Light: Science & Applications, Anlian Pan and colleagues at the Hunan University report a BP/CdS artificial photonic synapse with ultra-low power consumption^14^. The device shows a remarkable negative light response with maximum responsivity up to 4.1 × 10^8 ^A/W at V_D_ = 0.5 V and light power intensity of 0.16 μW/cm^2^ (1.78 × 10^8 ^A/W on average). Moreover, the device enables artificial synaptic applications with average power consumption as low as 4.78 fJ for each training process, representing the lowest among the reported results.

In this work, Anlian Pan and co-authors present an artificial photonic synapse structure based on BP/CdS van der Waals heterojunction (Fig. 1), where the CdS and BP are utilized as the photo-sensing layer and channel layer, respectively. Optical spikes are used as triggers thus driving the synaptic device. In this structure, a large number of electron-hole pairs will be generated in CdS flake with electrons flowing into BP and holes trapped in CdS due to the interface barrier between BP and CdS, and the defects and surface states in CdS. It should be pointed out that since both BP and CdS exhibit positive photoresponse, the negative photoresponse should be reasonably originated from the heterojunction caused by defect trapping or molecular adsorption.

**Fig. 1:**
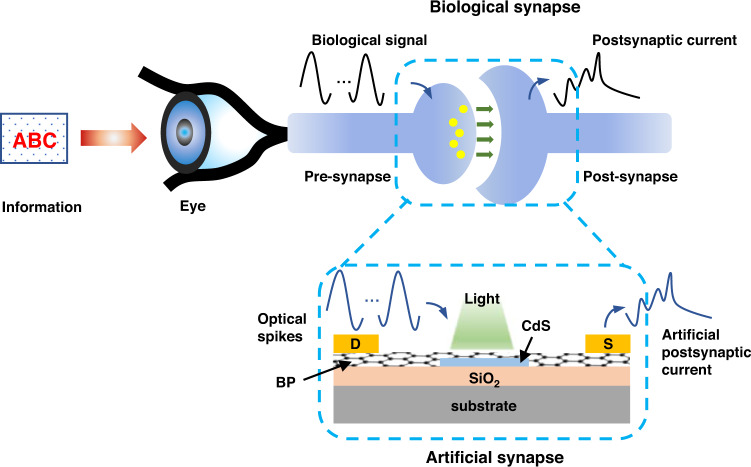
Schematic view of biological synapse and designed artificial synaptic device

This device exhibits good performance as follows: Firstly, it can be concluded that the responsivity of the BP/CdS HJ-FET is the highest among the reported devices so far^10–14^. Secondly, the trapped holes can be well maintained in CdS when the light illumination is removed, leading to a small current state being well kept in the channel. This charge storage behavior is similar to the long-term plasticity in biological synapse and is a prerequisite to ensure reliable study on the synaptic photoresponse. Thirdly, the results indicate that both light illumination time, intensity and different light pulse number can effectively modulate the synapse behavior, which enables the photonic synaptic device application.

With the presented work, the authors fabricate an artificial photonic synapse, by which typical photonic synaptic behaviors can be effectively modulated under the synergistic effect of light pulses and electrical pulses, including photosensitivity, postsynaptic photocurrents and persistent photoconductivity. They have also monitored the long-term potentiation (LTP) behavior of the synapse at 450 nm light pulse with different light stimulation information, including illumination time and intensity, revealing that longer light exposure time will effectively enhance the stimulus effect, leading to larger –ΔPSC. In addition, the -ΔPSC increases linearly with increasing the pulse number at first and then tends to saturate at a definite value with a fixed gate voltage. Thus LTP and electrical-response-driven long-term depression are successfully simulated based on the synergistic effect of optical programming and electrical erasing in the artificial synapse. The results reveal that the device can be switched well between the program and erase state with >150 cycles over 3000 s. Moreover, the authors simulate the potentiation and depression processes continuously by applying consecutive light and VG spikes, reflecting repeatable switching and good endurance performance of the device. Furthermore, a fully connected optoelectronic neural network (FONN) based on the typical optoelectronic synaptic behavior of the artificial photonic synapse is constructed to evaluate the accuracy of image recognition for the Modified National Institute of Standards and Technology (MNIST) handwriting image dataset. The results show that maximum recognition accuracy of 94.1% can be achieved after training.

The artificial photonic synapse exhibits photonic potentiation and electronic depression behaviors, indicating that the artificial synaptic devices support optical-write and electronic-erase functions for learning and recognition in artificial neural networks. This study provides a new concept for the designing of energy-efficient artificial photonic synapses and shows great potential in high-performance neuromorphic vision systems. We can expect that the performance of the proposed scheme can be further promoted by reducing power consumption of the photonic synapse, improving the recognition accuracy to higher sensitivity and increasing the device integration, this device will be widely applied for the development of photonic synaptic devices including memristors, field-effect transistors, and phase change memory^15–17^. Therefore, it will provide a promising application of 2D heterojunctions for neuromorphic computation, machine vision and artificial intelligence systems.
